# Synergistic and Additive Effects of Epigallocatechin Gallate and Digitonin on *Plasmodium* Sporozoite Survival and Motility

**DOI:** 10.1371/journal.pone.0008682

**Published:** 2010-01-13

**Authors:** Janina K. Hellmann, Sylvia Münter, Michael Wink, Friedrich Frischknecht

**Affiliations:** 1 Department of Infectious Diseases, University of Heidelberg Medical School, Heidelberg, Germany; 2 Department of Biology, University of Heidelberg, Heidelberg, Germany; BMSI-A*STAR, Singapore

## Abstract

**Background:**

Most medicinal plants contain a mixture of bioactive compounds, including chemicals that interact with intracellular targets and others that can act as adjuvants to facilitate absorption of polar agents across cellular membranes. However, little is known about synergistic effects between such potential drug candidates and adjuvants. To probe for such effects, we tested the green tea compound epigallocatechin gallate (EGCG) and the membrane permeabilising digitonin on *Plasmodium* sporozoite motility and viability.

**Methodology/Principal Findings:**

Green fluorescent *P. berghei* sporozoites were imaged using a recently developed visual screening methodology. Motility and viability parameters were automatically analyzed and IC50 values were calculated, and the synergism of drug and adjuvant was assessed by the fractional inhibitory concentration index. Validating our visual screening procedure, we showed that sporozoite motility and liver cell infection is inhibited by EGCG at nontoxic concentrations. Digitonin synergistically increases the cytotoxicity of EGCG on sporozoite survival, but shows an additive effect on sporozoite motility.

**Conclusions/Significance:**

We proved the feasibility of performing highly reliable visual screens for compounds against *Plasmodium* sporozoites. We thereby could show an advantage of administering mixtures of plant metabolites on inhibition of cell motility and survival. Although the effective concentration of both drugs is too high for use in malaria prophylaxis, the demonstration of a synergistic effect between two plant compounds could lead to new avenues in drug discovery.

## Introduction

Plant-derived bioactive compounds are attractive candidates for drug development since they represent lead structures for new or existing drug targets. Currently, most medications including antibiotics, anticancer drugs and drugs directed against parasites are based on natural compounds [1], [2], [3]. The active substances in plants are mostly secondary metabolites, which are used as defense compounds against predators or parasites, for interspecies competition or as signal compounds to attract insects or other animals [4]. Plants usually produce and store complex mixtures of compounds from different structural classes to simultaneously interfere with several targets, which is thought to amplify their efficiency and reduce the risk of resistance development by pathogens [5]. While many classes of secondary metabolites interfere with specific molecular targets members of one major secondary metabolite class called polyphenols interact non-selectively like “protein glue” affecting any protein they encounter [1], [2]. Thus, they influence amongst others catalysis, transport, ion exchange or signal transduction pathways of the targeted organism [6], [7].

Polyphenols can be divided in different families, for example the flavonoids, which in turn include the group of catechins [5]. The epigallocatechin gallate (EGCG) is the most effective and best-studied catechin in green tea [8], [9], [10]. EGCG was shown to generally inhibit metallo- and serine-proteases, proteasome function, cell cycle regulation, tumor invasion, and to reduce oxygen-derived free radicals [11], [12], [13]. Further, antibacterial effects as well as inhibition of human immunodeficiency virus (HIV) infection by EGCG were demonstrated [14], [15]. Another class of secondary plant metabolites are the so called terpenoids that contain a family of steroids including the group of saponins [16]. These saponins are together with polyphenols common in most higher plants, being a highly heterogeneous group of steroidal and triterpene glycosides [7], [16], [17]. Saponins are amphiphilic compounds that can interact with biomembranes, lyse cells or can form complexes with cholesterol. This interaction influences membrane fluidity and renders membranes leaky. Due to their pore forming activity anti-cancer effects of saponins as well as synergistc effects as (vaccine) adjuvants for substances that are normally not absorbed were also described [16], [18], [19]. The saponin digitonin, an amphipathic, membrane-disrupting steroid, is obtained from *Digitalis purpurea* [16].

Potential antimalarial properties of EGCG were also studied. It has been shown that EGCG binds to the intercellular adhesion molecule 1 (ICAM 1) on the endothelium thus blocking the adhesion of *Plasmodium* infected erythrocytes [20]. EGCG further inhibits *Plasmodium falciparum* growth *in vitro* and potentiates the antimalarial effects of artemisinin [21]. Finally, the mechanism of inhibition of an enoyl-acyl carrier protein reductase of *Plasmodium falciparum* (PfENR) by EGCG was investigated resulting in the inhibition of the fatty acid biosynthesis in the parasite [10].

*Plasmodium* sporozoites are the forms of the malaria parasite injected into the host during a blood meal of the mosquito [22]. Sporozoites are deposited within the skin, where they can move at a speed of around 1 µm/s [23], [24]. Some sporozoites can enter blood vessels and are transported to the liver, where they invade hepatocytes to differentiate into red blood cell invading merozoites [24], [25]. While most efforts to design interventions against malaria are focused on the blood stage of the parasite, which is responsible for the symptoms of the disease, a number of studies provide a good rationale for also focusing on the pre-erythrocyte stages [25]. However little effort is currently devoted to develop drugs against these pre-erythrocyte stages of the malaria parasite, which could prevent infection. We recently developed a methodology that should in principle allow visual screening for substances that inhibit sporozoite motility [26]. In order to verify this methodology, we probe here the effect of EGCG in combination with the adjuvant digitonin as a membrane permeabilising agent on *Plasmodium* sporozoite motility and survival.

## Results

### Effect of EGCG on *Plasmodium* Sporozoite Survival, Motility, and Liver Cell Infection

**Figure 1:**
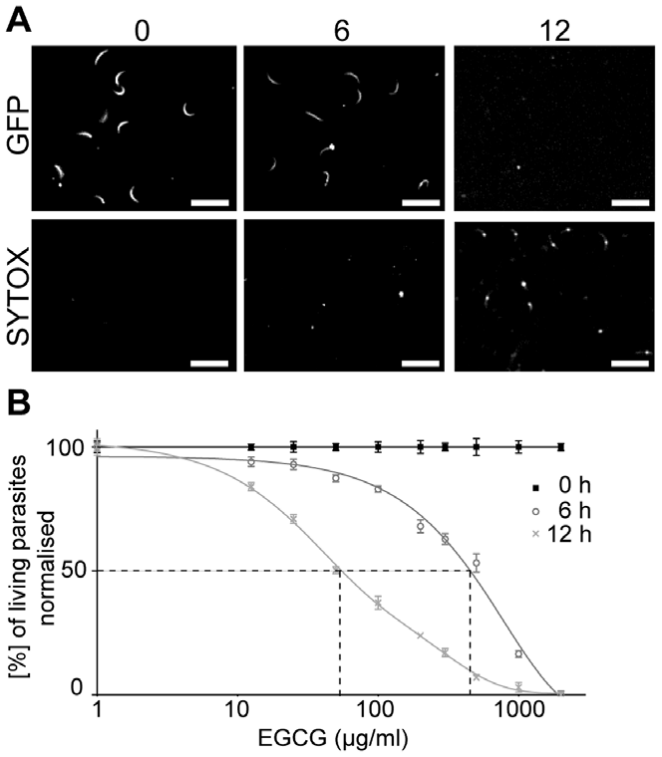
Cytotoxicity of EGCG on *Plasmodium* sporozoites. The percentage of living parasites after application of different EGCG concentrations from 12.5 µg/ml to 2000 µg/ml (27 µM - 4400 µM) was determined over 18 h. **A)** Snap shots of parasites after treatment with 200 µg/ml EGCG at the start of the experiment and after 6 and 12 h. The upper panels show the green, the lower panels the red fluorescent signal. Living parasites are crescent shaped and show green fluorescence. Dying parasites are round and exhibit red fluorescence. Scale bar: 25 µm; numbers indicate time in hours. **B)** The percentage of living parasites (normalised to the control set to 100%) was plotted over the concentrations of EGCG at 0 h (filled squares), after 6 h (open circles) and 12 h (crosses). Weighted averages of the triplicates (each usually contained between 200 and 300 sporozoites) and standard errors of the mean of the percentage of living parasites for each data point are shown. The dashed lines mark the IC50 values: 502±2 µg/ml (1095±3 µM) after 6 h, 54±2 µg/ml (118±4 µM) after 12 h.

We first performed a cytotoxicity assay over 18 h with EGCG concentrations ranging from 12.5 µg/ml to 2000 µg/ml (27 µM to 4400 µM) by imaging *Plasmodium* sporozoites expressing the green fluorescent protein (GFP). Living parasites were distinguished by their cytoplasmic green fluorescence from dead parasites that lost their GFP signal, but were red fluorescent after the uptake of SYTOX-Orange. When no drugs are added about 50% of the parasites died during the 18 h time course. Treatment with 200 µg/ml EGCG showed that parasites first rounded up, died and could then be found in the red channel (Figure 1A). After 6 h the first dead parasites were detected and after 12 h nearly all parasites were dead. EGCG concentration curves for different incubation times revealed a dose-dependent decrease of living parasites and yielded IC50 values of 502±2 µg/ml (1095±3 µM) and 54±2 µg/ml (118±4 µM) for 6 h and 12 h incubation periods, respectively (Figure 1B).

**Figure 2:**
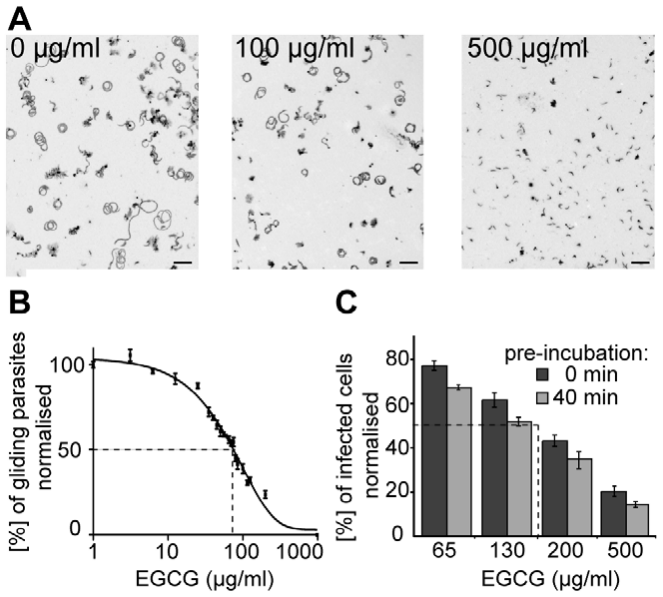
Inhibition of gliding motility by EGCG. The percentage of gliding parasites after treatment with EGCG in a range from 3.125 µg/ml to 500 µg/ml (6.75 µM to 1090 µM) was determined. **A)** Inverted maximum projections of time-lapse movies from untreated sporozoites and sporozoites treated with 100 µg/ml or 500 µg/ml. As sporozoites glide in circles, maximum projections of motile parasites show up as circles. At 100 µg/ml more than 50% of gliding was inhibited; at 500 µg/ml no motility was detected. Scale bar: 25 µm. **B)** Concentration dependent inhibition of parasite gliding motility after 40 min of incubation with EGCG. Weighted averages of triplicates (each usually contained between 200 and 300 sporozoites) and standard errors of the mean of the percentage of motile parasites were calculated for each concentration point, normalised to the control (set to 100%) and plotted over the applied EGCG concentrations. IC50 value: 63±1 µg/ml (137±1 µM). **C)** Inhibition of hepatocyte infection of sporozoites in the presence of various concentrations of EGCG. Sporozoites were either pre-incubated for 40 minutes in EGCG containing medium before addition to hepatocytes (light grey) or not pre-incubated (dark grey). The dashed black lines mark the IC50 which is aorund 130 µg/ml after pre-incubation.

To test for a possible effect of EGCG on sporozoite gliding motility, sporozoites exposed to EGCG concentrations ranging from 3.125 to 500 µg/ml (6.75 to 1090 µM) were imaged 40 min after incubation with the drugs. Maximum fluorescent intensity projections of gliding parasites showed that gliding was inhibited to around 50% and 100% after application of 100 µg/ml or 500 µg/ml, respectively (Figure 2A). Analysis of the time lapse movies with a recently developed tracking tool revealed a dose-dependent inhibition of the gliding motility by EGCG with an IC50 value of 63±1 µg/ml (137±1 µM) (Figure 2B). At this concentration and time point no effect of EGCG on sporozoite viability was detected. Only after more than 5 h of incubation a small lethal effect with ∼20% of sporozoites dying could be detected for this concentration. We next tested the effect of EGCG mediated inhibition of sporozoite gliding on liver cell infection. Sporozoites treated with different EGCG concentrations were either directly added to Huh7 cells or after an incubation period of 40 min (pre-incubation). This showed a dose dependent effect of EGCG on liver stages (Figure 2C). As expected the effect of inhibition was larger when sporozoites were pre-incubated with EGCG before adding them to the liver cells. Curiously, the IC50 for inhibition of liver cell infection was about two-fold higher as the IC50 for inhibiting sporozoite gliding.

### EGCG and Digitonin Act Synergistically to Kill Sporozoites

**Figure 3:**
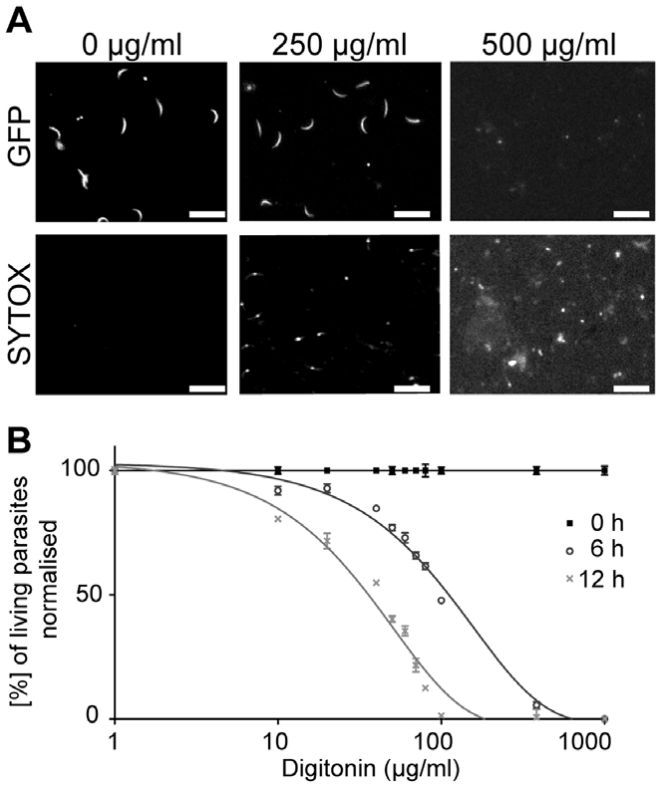
Permeabilisation efficiency of digitonin. **A)** Snap shots of non-treated sporozoites and sporozoites treated with 250 and 500 µg/ml digitonin after 40 min of incubation. The upper panels show the green, the lower panels the red fluorescent signal. Scale bar: 25 µm. **B)** The percentage of living parasites (normalised to the control set to 100%) plotted over the concentrations of digitonin at the beginning of the experiment (filled squares), after 6 h (open circles) and 12 h (crosses). Weighted averages of triplicates (each usually contained between 200 and 300 sporozoites) and standard errors of the mean are shown.

While the effect of EGCG on parasite gliding could be due to the unspecific binding and blocking of proteins at the parasite surface, the effect on viability could additionally be mediated by interactions of EGCG with intracellular targets. Since EGCG does not effectively cross the plasma membrane of the sporozoites, we next determined possible membrane permeabilising effects of the saponin digitonin. Snap shots of a pre-screen show that a digitonin concentration of 500 µg/ml permeabilised the membrane and killed the parasites within 40 min (Figure 3A). Reduction of the digitonin concentration to 250 µg/ml showed that more than 50% of the parasites are still alive after treatment (Figure 3A). Cytotoxicity assays were then also performed for digitonin concentrations in a range from 10 µg/ml to 1000 µg/ml over 18 h. The application of digitonin on sporozoites revealed a dose- and time-dependent decrease of living parasites (Figure 3B). After 6 (12) h at 60 µg/ml digitonin 28% (52%) of sporozoites respectively were killed.

**Figure 4:**
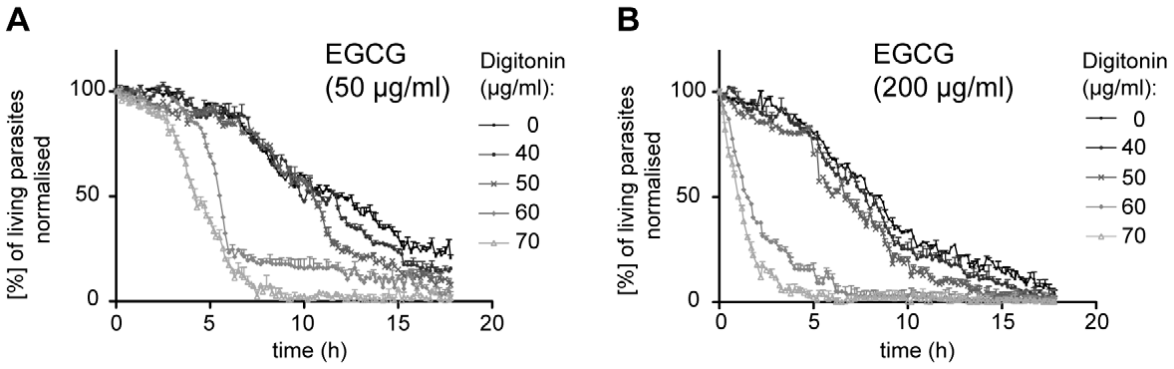
Cytotoxicity of EGCG combined with digitonin. The percentage of living parasites (normalised to the control set to 100%) after exposure to a low **A)** or high **B)** concentration of EGCG, combined with digitonin in a range from 40 to 70 µg/ml (40 = grey line with filled circle; 50 = grey line with cross; 60 = light grey line with filled rhombi; 70 = light grey line with open triangle) decreases with time. The values were averaged over triplicates (each containing 200 to 300 parasites) and normalised to the cytotoxicty assay of 40 µg/ml to 70 µg/ml digitonin without EGCG application measured over 18 h since digitonin alone showed a lethal effect (not shown, but partially included in Figure 3).

**Figure 5:**
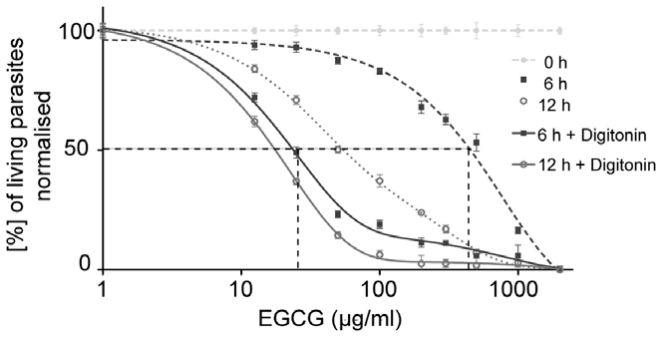
Synergistic cytotoxicity of EGCG and digitonin. The percentage of living parasites over (normalised to the control set to 100%) the concentrations of EGCG (dashed grey lines, already shown in Figure 1) and EGCG with 60 µg/ml (115 µM) digitonin was plotted at the beginning of the experiment (straight line on top), after 6 h (without digitonin = dashed black line with filled squares; with digitonin = black line with filled squares) and 12 h (without digitonin = dashed grey line with open circles; with digitonin = grey line with open circles). Weighted averages of triplicates (each usually contained between 200 and 300 sporozoites) and standard errors of the mean are shown normalised to the death curves of digitonin alone. The dashed black lines mark the IC50 values. The IC50 values of the cytotoxic effect of EGCG are reduced from 502±2 µg/ml (1095±3 µM) to 20±1 µg/ml (44±2 µM) after 6 h and from 54±2 µg/ml (118±4 µM) to 16±1 µg/ml (35±2 µM) after 12 h by 60 µg/ml digitonin application.

To determine possible additive or synergistic effects we next performed cytotoxicity assays over 18 h after application of EGCG concentrations from 12.5 µg/ml to 1000 µg/ml together with either 40, 50, 60 or 70 µg/ml digitonin. The data was normalised to the measured death curves of 40, 50, 60 and 70 µg/ml digitonin alone over 18 h since digitonin alone already had a toxic effect (data not shown). Death curves at a fixed EGCG concentration of 50 µg/ml or 200 µg/ml indicated that the toxic effect of EGCG was increased by digitonin in a dose-dependent manner (Figure 4A, B). Addition of 40 µg/ml or 50 µg/ml digitonin resulted in similar concentration curves as application of EGCG alone during the first 10 h. Only after more than 10 h an effect of these digitonin concentrations was detected. A clear shift towards an increased toxic effect of EGCG by digitonin application was only reached by a combination of EGCG with 60 µg/ml or 70 µg/ml digitonin. Already after 4 h the percentage of living parasites rapidly decreased at a fixed EGCG concentration of 50 µg/ml (Figure 4A). At a higher EGCG concentration (200 µg/ml) the additional effect of digitonin was more pronounced and detected already after less than 2 h. The dose-response curves showed a clear time-dependent decrease of living parasites of the combination treatment after 6 h and 12 h (Figure 5). The IC50 value could be decreased by 96% from 502±2 µg/ml (1095±3 µM) for EGCG alone to 20±1 µg/ml (44±2 µM) in the presence of 60 µg/ml digitonin 6 h after application. The IC50 value after 12 h was also decreased by 70% from 54±2 µg/ml (118±4 µM) in the absence of digitonin to 16±0.84 µg/ml (35±2 µM) in the presence of 60 µg/ml digitonin. The calculated fractional inhibitory concentration ∑FIC of 0.25 demonstrated a toxic synergism between both drugs (for calculation see material and methods).

### No Synergistic Effects between EGCG and Digitonin on Sporozoite Motility

**Figure 6:**
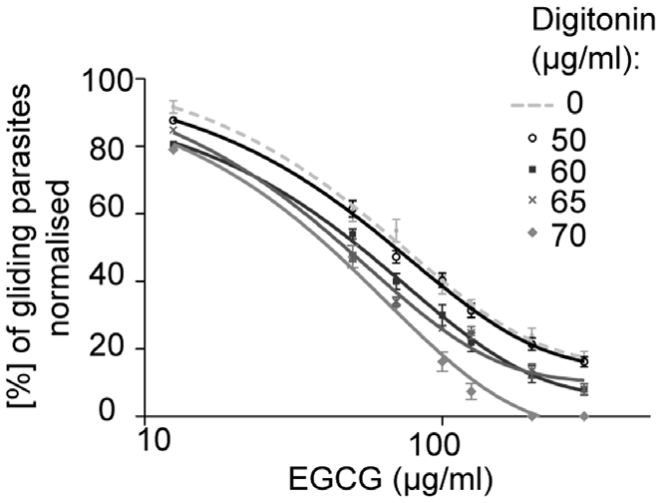
Inhibition of gliding motility by EGCG and digitonin. Concentration dependent inhibition of gliding sporozoites (normalised to the control set to 100%) treated with different EGCG concentrations and permeabilised with 50 (open circles), 60 (filled squares), 65 (crosses) and 70 (filled rhombi) µg/ml digitonin. IC50 value of EGCG and 60 µg/ml digitonin for inhibition of gliding motility: 52±1 µg/ml (113±2 µM). The EGCG curves based on 60 µg/ml and 65 µg/ml are similar, except of a sharper decline of the EGCG curve with 65 µg/ml digitonin.

We next tested for a possible synergistic effect of EGCG together with digitonin on the inhibition of sporozoite motility after 40 minutes. EGCG was applied over the entire concentration range together with 50, 60 and 70 µg/ml digitonin (Figure 6). The application of 50 µg/ml digitonin did not result in an increased effect of EGCG compared to the administration of EGCG alone. A significant shift of the EGCG curve was shown for EGCG in combination with 60 µg/ml digitonin. This parallel shift towards the curve of non-permeabilised parasites is typical for a synergistic effect of the adjuvant with the drug. The IC50 40 minutes after the application of the combination treatment was reduced by 20% to 52±1 µg/ml (113±2 µM) for the inhibition of gliding motility for a combination treatment with 60 µg/ml digitonin (IC50 for EGCG alone 63±1 µg/ml (137±1 µM)). The application of 70 µg/ml digitonin also caused a shift of the curve. However, the measurements revealed not only a motility inhibiting but also a lethal effect of 70 µg/ml digitonin in combination with EGCG doses over 100 µg/ml (Figure 6) (data not shown).

Since a shift of the EGCG curve was only achieved with a digitonin concentration between 60–70 µg/ml, an intermediate concentration of 65 µg/ml digitonin was applied together with EGCG (Figure 6). No parallel shift of the EGCG curve for 65 µg/ml digitonin compared to EGCG together with 60 µg/ml digitonin was detected. The percentage of gliding parasites was also similar for the 60 µg/ml and the 65 µg/ml dose of the adjuvant. Treatment with 65 µg/ml digitonin revealed a sharper decline of the regression concerning the EGCG range from 50–100 µg/ml suggesting an additive effect of digitonin. The calculated fractional inhibitory concentration ∑FIC of 0.82 demonstrated that there is no synergism between EGCG and digitonin concerning inhibition of gliding motility (for calculation see material and methods).

## Discussion

Here we tested (i) a recently developed assay for its utility in screening for inhibitors of *Plasmodium* sporozoite motility and (ii) for possible effects of the secondary plant metabolite EGCG together with the saponin digitonin on cytotoxicity and inhibition of motility. This revealed that such a combination of natural compounds results in a more efficient effect on a target cell than application of a single drug as monotherapy. While digitonin showed an additive effect to EGCG on inhibiting motility, it showed a synergistic effect on inhibiting sporozoite survival. However, EGCG inhibited the motility rapidly after 40 minutes, while causing sporozoite death only after several hours.

Most studies on the identification of potential drugs targeting malaria parasites are aimed at the blood stages of the parasites. So far, three studies demonstrated an antimalarial potential of EGCG on blood stages of *Plasmodium falciparum* [10], [20], [21]. To test the influence of EGCG on sporozoites, we performed cytotoxicity and motility assays. This revealed that EGCG inhibited the gliding motility of sporozoites in a time-dependent manner at concentrations where no toxic effect was detectable. Indeed, eight fold higher concentrations of EGCG were necessary to induce lethal effects on sporozoites than for inhibiting the motility (Figure 1 and 2). An EGCG concentration of 63 µg/ml caused a 50% inhibition of motility after 40 min (Figure 2). However, the same concentration of EGCG caused a 50% cytotoxic effect only after about 12 h (Figure 1). In the context of a natural infection, inhibition of sporozoite gliding in the skin or on the liver endothelium would be enough to stop the parasite from infecting the liver and thus interrupting its life cycle. Intriguingly, we found that a higher concentration of EGCG is needed to reduce liver cell infections to the same level as needed to reduce the number of motile sporozoites (Figure 2C). This suggests that EGCG has no detrimental effect on hepatocytes and indicates that gliding motility does not strictly correlate with successful infection. Indeed, it is well known that sporozoites migrate through a number of cells before finally invading a hepatocyte for differentiation into merozoites [27]. Thus efficient gliding might be more relevant for migration in tissue or through cells than for invasion.

But how does EGCG inhibit motility and cause cytotoxicity? EGCG is a polyphenol, which can form several hydrogen and ionic bonds with proteins, thus modulating their 3D structure [1], [2]. At physiological pH, the eight phenolic hydroxyl groups of EGCG are partly dissociated. In our assay, we hypothesise that EGCG binds to adhesion molecules on the parasite surface, e.g. TRAP or CSP that are important for motility and thus impair gliding [28], [29]. The non-covalent bonds could lead to an inactivation of the surface proteins and the parasites could thus be rendered immotile. Such unspecific effects are suggested by the high IC50 value (µM range instead of nM range for specific interactions). However, some authors suggested direct functional inhibitory interactions of EGCG even at similarly high concentration (200 µM) [12], [20]. Alternatively, EGCG could also interact with other proteins in the medium, for example BSA, which is needed for efficient gliding motility. If EGCG would bind to BSA and in addition on the sporozoite, a quenching effect of BSA could explain the observed high concentration of EGCG needed for inhibition of motility. However, the data in Figure S1 show that decreasing the BSA concentrations from 1% to 0.25% does not change the effect of EGCG significantly. Only at higher concentrations of BSA quenching was observed (Figure S1). Clearly, even higher EGCG concentrations may be necessary to achieve a sporozoite effect *in vivo*. Extrapolating from the *in vitro* assay, EGCG might *in vivo* not only bind to sporozoite adhesion proteins on the surface of the parasites but also to receptors on the host cell or proteins within the tissue. Many side effects could further follow the application of EGCG since the polyphenol inhibits different proteins non-selectively. We therefore reasoned that the application of an adjuvant could increase the effect of EGCG on target cells, decrease the necessary efficient dosage of EGCG and thus reduce adverse reactions. This could be achieved in different ways causing either a simple additive or a synergistic effect. Using the membrane permeabilising saponin digitonin, we measured digitonin applications of different concentrations in cytotoxicity assays over 18 h alone to normalise the data of the combination treatment of EGCG and digitonin against the toxic effect the adjuvant already determines alone. After normalisation, we observed an additional effect on inhibition of gliding motility and, importantly, a synergistic effect on parasite killing. Amphiphilic saponins have detergent properties that can disrupt membrane fluidity, inhibit the function of membrane proteins such as ion channels and disrupt the membrane [1], [2]. Therefore, saponins can exert either an additive or synergistic effect by disturbing the cell membrane or promoting the transport of EGCG.

Consequently, digitonin could also enhance in both possible ways the effect of EGCG on gliding motility. For example, a membrane disrupting effect could potentially lead to an additional effect of EGCG on the parasite motor machinery, which is localised underneath the plasma membrane. Intracellular proteins involved in motility could also be targeted by EGCG [30]. The mode of action is non-synergistically since our data leads to a calculated ∑FIC of 0.82. Based on the guideline paper for the FIC index by Hall et al. we suggest that digitonin enhances the action of EGCG on gliding motility rather by a dominant additive effect [31].

However, a clear synergistic effect (∑FIC = 0.25) of EGCG with digitonin on the inhibition of sporozoite survival was shown. It is likely that besides causing membrane disruption digitonin promotes the transport of EGCG inside the parasite. Due to the later effect of EGCG, the polyphenol might enter over time he parasite to exert the toxic effect. EGCG might also induce signalling pathways from the cell surface that lead to cell death. The high concentration necessary to inhibit motility and the late action on sporozoite survival along with the low bioavailability [32] (several tens of litres of green tea would have to be consumed to reach inhibitory concentrations in the blood) show that EGCG itself can not be developed into an anti-malarial drug. However, both compounds can be considered as lead structures for drugs against other ailments.

Importantly, we could demonstrate that the motility apparatus of sporozoites can be in general targeted by drugs. Although our tested combination will not be useful for fighting malaria, our results validate a recently developed visual screening method for drug discovery based on automated analysis of sporozoite motility [26]. This assay could thus lead to the discovery of compounds that inhibit or kill the malaria parasite before it even enters the first host cell for replication.

In conclusion, by using a new visual screening approach we could show the advantage of plant metabolites mixtures resulting in a cytotoxic synergistic effect on a pathogen. The fact that such synergism was found suggests new avenues in screening for inhibitory molecules and the development of multitarget drug cocktails.

## Materials and Methods

### Preparation of *Plasmodium* Sporozoites

*Plasmodium berghei* (strain NK65) sporozoites constitutively expressing cytoplasmic green fluorescent protein (GFP) under the control of the stage specific circumsporozoite (CS) promoter were produced in *Anopheles stephensi* mosquitoes. Therefore, *Anopheles stephensi* mosquitoes (Sda500 strain) were raised at 28°C, 75% humidity, under a 13/11 h light/dark cycle and maintained on 10% sucrose solution and 10 µg para-aminobenzoic acid in a standard insectary. NMRI mice (Charles River) were infected by intraperitoneal injection of 200 µl frozen parasite stock. All animal experiments were performed concerning FELASA category B and GV-SOLAS standard guidelines. Animal experiments were approved by the German authorities (Regierungspräsidium Karlsruhe, Germany), § 8 Abs. 1 Tierschutzgesetz (TierSchG).

The next 3–4 days Giemsa-stained (1∶10 solution, BDH) blood smears of the infected mice were analysed and the parasitaemia was determined. At a sufficient parasitaemia the blood was gained by heart puncture and about 20 million parasites were transferred into a naïve mouse. Exflagellation of male gametocytes was checked three to four days later. If exflagellation occurred between 10–14 min with at least two events per field of view, the mouse was anaesthetized with 100–150 µl 10% ketamin/2% xylazin in PBS and fed to *Anopheles stephensi* mosquitoes (3–5 d after emergence). After infection the mosquitoes were maintained at 20°C and 80% humidity in standard incubators. 17–23 d after infection of the mosquitoes, the fluorescent sporozoites were isolated from infected salivary glands, and suspended in cold RPMI-1640 (PAA) supplemented with 1% bovine serum albumin (BSA, Roth).

Comparison of the gliding capacity of *P. berghei* sporozoites in medium containing various concentrations of BSA showed that sporozoites moved as robustly in medium containing 1% BSA than in medium containing 3% BSA. However, at 0.5% and 0.25% BSA the number of motile sporozoites dropped drastically (Figure S1A). We therefore performed all experiment in the presence of 1% BSA. Indeed no difference of the effect of EGCG on sporozoite motility was found between media containing 1%, 0.5% and 0.25% BSA (Figure S1B). But the reliability of movement decreased with decreasing BSA concentrations as evidenced by the increased standard deviation (Figure S1B).

### Cytotoxicity and Motility Assays

A stock solution of 4 mg/ml (8726 µM) EGCG was prepared in RPMI and diluted to EGCG concentrations ranging from 12.5 µg/ml to 2000 µg/ml (27 µM to 4400 µM) to measure dose response curves. Further a stock solution of 2 mg/ml (1627 µM) digitonin was prepared in RPMI and diluted to concentrations ranging from 10 µg/ml to 1000 µg/ml (8 µM to 813 µM).

In every experiment 8–10 different conditions were measured for both, the cytotoxicity or motility assay. For both assays 23 µl of the medium containing isolated sporozoites were placed in one well of a 384 well plate (with optical glass bottom), which was then centrifuged at 375 g for 5 min to sediment the parasites. Following, 25 µl of EGCG (Sigma) or digitonin (Sigma) were mixed with 2 µl SYTOX-Orange (100 µM stock, Invitrogen) and added to the parasite solution. SYTOX-Orange binds to the DNA of permeabilised parasites and is therefore used as a marker to generate death curves in the cytotoxicity assay (life-dead assay).

### Microscopy and Image Analysis

For cytoxicity assays the total number of living (green fluorescence) and dead (red fluorescence, SYTOX-Orange) parasites present in each field of view was counted over 18 h with images of both channels taken every 10 min. For motility assays sporozoites were incubated for 40 min with the different drug concentrations at room temperature. Following, images were collected with a time lapse of 0.5 Hz for 100 frames in the green channel. To analyse whether all parasites were still alive in the motility assays a short time lapse of the SYTOX-Orange signal (red channel) and the GFP signal was taken before and after 100 frames. Imaging was performed at room temperature (24°C) in an air-conditioned imaging suite with an inverted Axiovert 200M Zeiss microscope using a GFP filterset to detect green fluorescence (450 nm/510 nm) [26]. The red fluorescent SYTOX-Orange signal was detected with a RFP filter set (546 nm/575–640 nm). Images were collected with a Zeiss Axiocam HRM using the Axiovision 4.6 software and a 10x Apoplan objective (NA = 0.25).

For each well three different regions of interest (ROI) were recorded to yield triplicates. Each ROI usually contained between 200 and 300 sporozoites. Thus, approximately 700 sporozoites were imaged for every concentration in one experiment. Every experiment was repeated at least three times. All image series were imported to ImageJ for analysis. For life dead assays the total number of green fluorescent, living parasites was automatically counted applying a custom-made ImageJ plugin (Hegge et al., in preparation). For motility assays the movies were analysed with regard to the speed (µm/s) of gliding parasites by applying the ImageJ plugin ToAST [26]. In brief, after background subtraction and application of an automatic threshold, the plugin automatically processes the data, detects the different motility patterns and calculates the gliding speed. Weighted averages and weighted standard errors of the mean (SEMs) were calculated in percent and normalised to the control set to 100%.

### Infection Assay

To test the effect of inhibiting sporozoite motility on hepatocyte infection we cultivated Huh7 cells in Dulbecco's modified Eagle's medium supplemented with 10% fetal calf serum and antibiotics. Sporozoites dissected in RPMI with 1% BSA and treated with various concentrations of EGCG were added to the cells in two different ways. Sporozoites were either directly added to the cells or pre-incubated for 40 minutes at room temperature before being added to the hepatocytes. Liver cell infection was performed in a similar way as described in Silvie et al [33]. Cultivated in 8-chamber plastic Lab-Tek (Thermo Fisher Scientific, nunc) slides the Huh7 cells were incubated with the sporozoites (10.000 for each condition) treated with 0, 65, 130, 200 or 500 µg/ml EGCG for one hour at room temperature and two hours at 37°C. Assays were performed in duplicates (without pre-incubation) and in triplicates (with pre-incubation). After 48 h, exoerythrocytic forms were revealed using primary antibodies against *Plasmodium* heat shock protein 70, followed by the anti-mouse Alexa Fluor 488 secondary antibody. DAPI (Sigma) was used to stain nuclei. Images were acquired with the same microscopic setup as described earlier. Infected hepatocytes were manually counted and noted as percentage of the total hepatocytes after 48 h of incubation. The percentage values were then normalised to the control wells of liver cells infected with non-treated sporozoites.

### Calculation of IC50 and ∑FIC

The graphs, logarithmic regression and IC50 values were generated and calculated using GraphPrism3 (GraphPad Software). Synergism of drug and adjuvant was evaluated by calculating the fractional inhibitory concentration index (∑FIC) as follows: ∑FIC = (minimal inhibitor concentration (MIC) of combination A + B)/(MIC of drug A alone) + (MIC of combination A + B)/(MIC of drug B alone) [34]. The unit of the MIC values is mg/l. A synergistic effect is indicated if the FIC index is smaller than 0.5 (significance level). The MIC values were defined as the concentrations when 10% of the parasites were dead or when 10% of the gliding motility was inhibited.

### Calculation of ∑FIC for Sporozoite Viability Studies

The cytotoxic synergism between EGCG and digitonin was evaluated by calculating the fractional inhibitory concentration index (∑FIC). For EGCG ( = A) a MIC value of 36 µg/ml (79 µM), for digitonin ( = B) a MIC value of 22 µg/ml (18 µM) and for the combination of the whole EGCG concentration range with 60 µg/ml digitonin (A+B) a MIC value of 3 µg/ml (6.5 µM) was determined from the death curves after 6 h (Figure 1B, 3B).

### Calculation of ∑FIC for Inhibition of Motility

The ∑FIC was calculated for the inhibition of motility by EGCG combined with digitonin. For EGCG ( = A) a MIC value of 15 µg/ml (33 µM), for Digitonin ( = B) a MIC value of 60 µg/ml (49 µM) and for the combination of the whole EGCG concentration range with 60 µg/ml Digitonin (A+B) a MIC value of 9 µg/ml (20 µM) was determined from the inhibition curves after 40 minutes (Figure 2B, 3B).

## Supporting Information

Figure S1 Gliding motility of sporozoites and bovine serum albumine. A) The percentage of gliding parasites depends on the concentration of bovine serum albumine (BSA) concentration. Normally gliding studies are performed with 3% BSA. We reduced it to 1% since the percentage of sporozoite gliding is still similar. However, decreasing the BSA concentration further resulted in a drastic reduction of gliding parasites. In addition, the standard deviation increases with decreasing BSA concentrations. B) Epigallocatechin gallate (EGCG) inhibits gliding of sporozoites. No change in the efficiency of EGCG inhibition was found for sporozoites gliding in the presence of 0.25%, 0.5%, or 1% of BSA. However, in the presence of 3% BSA, the efficiency of inhibition was decreased as markedly more parasites were gliding. (*: p<0.05; n.s. = not significant)
(0.13 MB TIF)
