# Supplementary figures and images for: Synergistic and Additive Effects of Epigallocatechin Gallate and Digitonin on Plasmodium Sporozoite Survival and Motility
Source: PLoS One. 2010 Jan 13;5(1):e8682. doi: 10.1371/journal.pone.0008682 (PMC2800191; doi:10.1371/journal.pone.0008682)

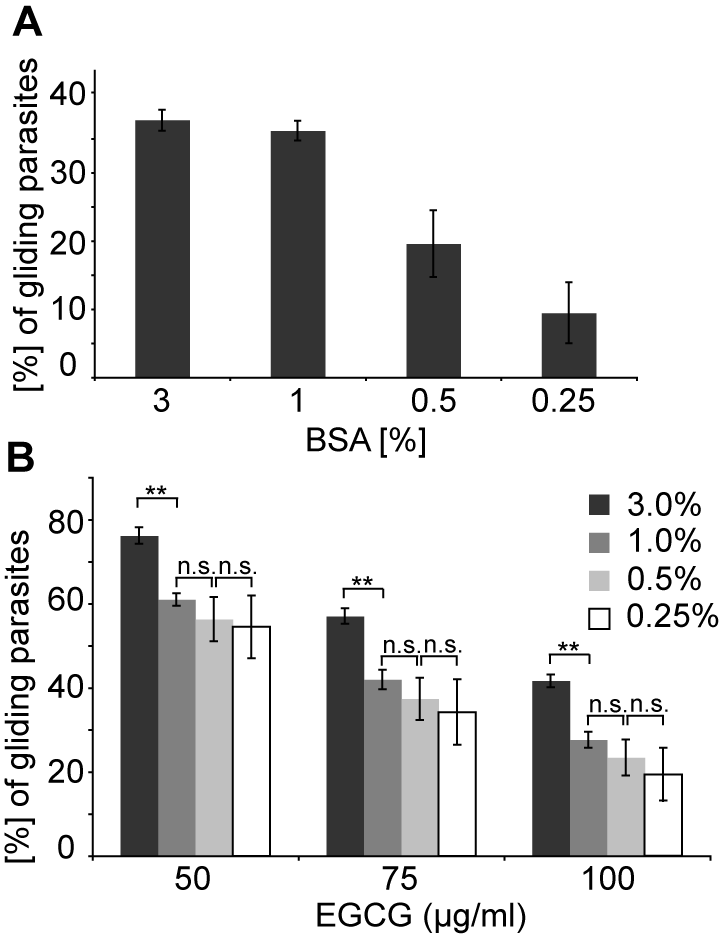

Supplement: Figure S1 — Gliding motility of sporozoites and bovine serum albumine. A) The percentage of gliding parasites depends on the concentration of bovine serum albumine (BSA) concentration. Normally gliding studies are performed with 3% BSA. We reduced it to 1% since the percentage of sporozoite gliding is still similar. However, decreasing the BSA concentration further resulted in a drastic reduction of gliding parasites. In addition, the standard deviation increases with decreasing BSA concentrations. B) Epigallocatechin gallate (EGCG) inhibits gliding of sporozoites. No change in the efficiency of EGCG inhibition was found for sporozoites gliding in the presence of 0.25%, 0.5%, or 1% of BSA. However, in the presence of 3% BSA, the efficiency of inhibition was decreased as markedly more parasites were gliding. (*: p<0.05; n.s. = not significant) (0.13 MB TIF) [file pone.0008682.s001.tif]
